# Mercury Determination in Natural Zeolites by Thermal Decomposition Atomic Absorption Spectrometry: Method Validation in Compliance with Requirements for Use as Dietary Supplements

**DOI:** 10.3390/molecules24224023

**Published:** 2019-11-06

**Authors:** Marin Senila, Oana Cadar, Lacrimioara Senila, Alexandra Hoaghia, Ion Miu

**Affiliations:** 1 National Institute for Research and Development of Optoelectronics Bucharest INOE 2000, Research Institute for Analytical Instrumentation, 67 Donath Street, 400293 Cluj-Napoca, Romania; oana.cadar@icia.ro (O.C.); lacri.senila@icia.ro (L.S.); alexandra.hoaghia@icia.ro (A.H.); 2 SC UTCHIM SRL, 12 Buda Street, 240127 Ramnicu Valcea, Romania; utchim_vl@yahoo.com

**Keywords:** mercury, zeolites, TD-AAS, method validation, green methods, dietary supplements

## Abstract

Natural zeolites are hydrated aluminosilicate minerals that, due to their remarkable physical-chemical properties of being molecular sieves and cation exchangers, have applications in different areas such as environmental protection, catalysis, animal feed, and dietary supplements. Since natural zeolites may contain traces of undesirable compounds such as toxic metals, the accurate quantification of these elements is necessary. In this study, a direct method for Hg determination in zeolite samples based on the thermal desorption atomic absorption spectrometry (TD-AAS) technique is fully validated, taking into account the legislative requirements in the field. The chosen quantification limit was 0.9 µg kg^−1^, which is satisfactory for intended use. Trueness was evaluated by recovery rate using certified reference materials containing mercury, with satisfactory results. Other figures of merit, such as repeatability and measurement uncertainty, also fulfill the legislative requirements related to the analysis of dietary supplements. This paper presents, for the first time, a fully validated method for mercury determination in zeolite samples, and the obtained results reveal that the method can be applied successfully for the intended purpose.

## 1. Introduction

Zeolites are crystalline aluminosilicate minerals that belong to the class of tectosilicates that comprise about 75% of the Earth′s crust. They have a three-dimensional cage-like structure of SiO_4_ and AlO_4_ tetrahedra, with well-defined channels and cavities [1,2]. Zeolites’ structure has a net negative charge that is balanced by exchangeable cations (sodium, potassium, and calcium) [3]. Due to their structure, zeolites have remarkable properties: adsorption capacity, cation exchange, dehydration-rehydration, molecular sieve ability, and catalysis features, and, as a result, they have found extensive application in environmental protection, chemistry, biotechnology, water treatment, agronomy, and medical areas [4,5,6].

Zeolites occur naturally, but can also be synthesized in the laboratory. Usually, natural zeolites are formed by the transformation of volcanic rocks (tuff) in contact with fresh water or seawater [7]. Recently, naturally occurring zeolite materials, such as clinoptilolite, have been increasingly studied and used in veterinary and human medicine. Previous in vivo and in vitro studies revealed that the natural zeolites have positive effects on human health, being used mainly for detoxification [8]. Zeolites have an affinity for cations, which is an advantage for their use in removal of toxic metals from different media. On the other hand, even this capacity can lead to the accumulation, in naturally occurring zeolites, of some potentially toxic metallic ions at the trace level [9]. Consequently, the use of natural zeolites for medical purposes as dietary supplements involves strict quality control.

Several recent papers have presented state-of-the-art methods for chemical analysis for the control of contaminants in foodstuffs, food raw materials, and dietary supplements [10,11,12,13]. One of the elements that may occur naturally in zeolites, depending on their origin, is mercury (Hg). According to the World Health Organization (WHO), mercury is one of the most toxic chemicals that can have serious adverse impacts on human health, mainly affecting the central nervous system [14,15].

The development of analytical methods to determine low Hg levels in food (including dietary supplements), environmental and biological samples is of great interest [16,17,18,19,20,21,22]. Also, for Hg monitoring in the environment, several methods and devices have been developed for passive sampling. Chouhan and co-workers [23] have developed a nano-knitted green adsorbent to be used in passive sampler devices for Hg monitoring in water. Taking into account its high toxicity, and in order to protect human health, the Commission Regulation 629 of 2 July, 2008, amending Regulation (EC) No. 1881/2006 that sets maximum levels for certain contaminants in foods, established the maximum level of mercury content in dietary supplements at 0.1 mg kg^−1^ [24,25]. The accurate determination of this low value in solid samples represents a challenge for analytical chemistry. It should be taken into account also that Commission Regulation No. 333/2007 regarding the methods of sampling and analysis for the official control of the levels of lead, cadmium, mercury, inorganic tin, 3-MCPD, and benzo(a)pyrene in foodstuffs has established a set of performance criteria for the analytical methods applied in the laboratory for these compounds [26].

There are several analytical instrumental techniques that can be employed for mercury determination. Among them, inductively coupled plasma mass spectrometry (ICP-MS), atomic fluorescence spectrometry (AFS), and atomic absorption spectrometry (AAS) are the most used [27]. Commonly, in order to improve the selectivity and sensitivity of these instruments, their coupling with the cold vapor (CV) generation method is used [28], resulting techniques such as CV-ICP-MS, CV-AFS, and CV-AAS. The use of these techniques is time-consuming and requires chemical reagents. However, the sample introduction system of these methods are appropriate for liquid sample analysis, while for solid samples a preliminary step of sample digestion is necessary. This step is also time- and reagent-consuming and may lead to analyte loss or to sample contamination.

Thermal desorption atomic absorption spectrometry (TD-AAS) is a fast, reagent-free method that can be used for the determination of mercury directly in liquid and solid samples without a digestion step before instrumental analysis. TD-AAS method responds to the growing interest in developing green methods. The sample is decomposed in an oxygen stream when mercury is released and carried through a column of catalyst, being transformed into metallic mercury (Hg^0^), which is collected on a gold amalgamator. After the sample is entirely decomposed, the trapped mercury is released rapidly by fast heating the amalgamator, and transported to the detection system. This system consists of two measuring cells optimized for the measurement of low and high mercury concentrations, respectively, allowing the measurement of mercury using a large dynamic range for analysis, at different mercury concentrations. Through the two cells containing mercury atoms from the sample passes the light emitted at a wavelength of 235.7 nm by a mercury lamp as the source of a typical atomic absorption spectrometer. The initial intensity of radiation emitted by the source is reduced in a manner proportional to the number of Hg atoms in the cells, and is measured by the detector from the spectrometer [28,29,30].

Despite the increased use of natural zeolites for human health purposes in recent years [8], no literature data on the analytical methods developed for mercury determination in zeolites are reported. A standardized method, EPA Method 7473 [31], has been used as a reference method. To provide trustworthy analytical results, testing laboratories should validate their analytical methods by studying analytical performance parameters to demonstrate that the method is suitable for the intended use [32]. The literature is very scarce regarding information on the validation of methods for mercury determination in complex matrix samples.

The aim of this work was to perform a detailed validation of total Hg determination in zeolites using a commercial TD-AAS instrument. Considering the zeolite samples as a dietary supplement, in our study the demands of the Decisions 2007/333/EC, 2008/629/EC, 2006/1881/EC, and 2002/657/EC [33] on the determination of toxic elements in foods, including dietary supplements, were considered. In order to present the confidence interval of the results, an estimation of measurement uncertainty was performed. The method was applied to determine the total Hg content (particle bounded) in real zeolite samples collected from a deposit from Chilioara, north-west Romania. The paper is important for control analytical laboratories dealing with the determination of mercury in zeolites or even in other food supplements since it presents a fully-validated method for this purpose.

## 2. Results and Discussion

### 2.1. Method Validation

The validation of the analytical procedure for quantitative determination of mercury in zeolite samples was performed by evaluating selectivity, working and linear ranges, limit of detection (LoD) and limit of quantification (LoQ), trueness, precision, and measurement uncertainty. The performance parameters were compared with the requirements established by Commission Regulation No. 333/2007.

Several tests were performed to achieve the optimal instrumental parameters for zeolite sample analysis, and to reduce as much as possible the time of analysis; the targeted instrumental parameters were drying temperature and time, and decomposition temperature and time. Also, other parameters such as catalyst temperature, catalyst wait period, heating temperature of gold trap, measurement time, and oxygen flow rate were used. The instrumental settings used for the Hg analyzer for the all determinations are presented in Table 1.

#### 2.1.1. Selectivity

Selectivity refers to the ability of the technique to distinguish a particular analyte in a complex mixture without interference from other components [32]. The selectivity in the case of the TD-AAS technique is related to possible interferences of the absorbance spectrum at the specific wavelength of the Hg at 253.7 nm. The selectivity study revealed no significant changes in absorbance signal at this wavelength when empty combustion boats and combustion boats filled with ultrapure water, respectively, were introduced. Possible interferences for this technique are co-absorbing gases (some organics and free chlorine), but, due to the particularities of instrumentation, no interference should be present. In this technique, Hg is released from the sample and reduced to metallic mercury by a catalyst tube that acts also as a trap for other impurities. Subsequently, mercury vapors are retained on a gold trap by amalgamation, which is also a selective reaction for mercury [34]. By heating the gold trap to 700 °C, mercury is released and transported to a measurement cell where the measurement is done at a characteristic wavelength for mercury, 253.7 nm. All these aspects contribute to the good selectivity of the method.

#### 2.1.2. Working and Linear Ranges

**Figure 1:**
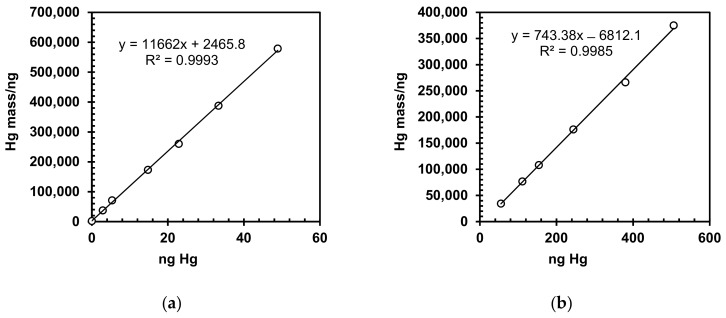
Calibration curves for Hg determination by TD-AAS: (**a**) for high sensitivity cell; (**b**) for low sensitivity cell.

The instrument was calibrated as a function of absorbance signal and mercury content. Two calibration curves were generated over the two ranges using a high sensitivity and a low sensitivity cell, respectively (Figure 1a,b). The correlation coefficients (r), 0.9993 and 0.9985, respectively, for both calibration ranges, fulfill the requirements of r > 0.995, demonstrating a good linearity.

For an independent check of calibration, a solution of 0.100 ± 0.010 mg L^−1^ Hg^2+^ was used, and the measured concentration was 0.107 mg L^−1^, which is a satisfactory result. The standard deviation for repeated measurements of this solution was lower than the target value of ± 10% (six parallel measurements). The lower part of the working range is the value of the limit of quantification (LoQ), while for the upper part, the critical parameter is linearity of the TD-AAS analyzer, which is up to 600 ng Hg. Thus, if a 100 mg sample is analyzed, the maximum concentration that can be analyzed is 600 mg kg^−1^.

#### 2.1.3. LoD and LoQ

LoD was estimated using the 3s criteria (Equation (1) by measuring the absorbance signal for 10 different blank solutions (5% HCl) using a calibration curve constructed using a high sensitivity range, according to Equation (1):

(1) $$\mathrm{LoD}=\frac{3\mathrm{SD}}{b}$$

where SD is the standard deviation for 10 parallel measurements of blank solutions and b is the slope of the calibration curve.

The LoD value, calculated by analyzing an amount of 100 mg sample, was 0.45 µ kg^−1^, while LoQ was considered to be two times LoD (0.90 µg kg^−1^). In order to check the LoQ value, a series of 10 spiked solutions with Hg content level of 0.90 µg kg^−1^ were analyzed. The relative standard deviation (%) was 15.6% and recovery in confirmation of the lower working range concentration was 109%, which is a satisfactory performance (the imposed targets were relative standard deviation of repeatability (RSDr) < 20% and a recovery in the range of 85–115%).

The LoD and LoQ method fulfills the requirements for Hg measurement in dietary supplements, since their values are at least 10 and 5 times, respectively, lower than the maximum admitted level of 100 µg kg^−1^ Hg (Commission Regulation 2006/1881/EC). 

#### 2.1.4. Trueness and Precision

Trueness is usually estimated by % recovery in certified reference materials (CRMs) analysis or by analysing spiked samples [35]. Six parallel samples of five different CRMs with a similar matrix to zeolite samples were analysed. The certified and measured values of CRMs, as well as their associated uncertainties, are presented in Table 2. 

Standard uncertainty associated with bias was calculated using Equation (2), i.e.,

(2) $$u(B)=\sqrt{B^{2}+u(C_{R})}$$

where B is bias from the certified value of CRM and *u*(*C_R_*) is the standard deviation of parallel measurements of CRM.

In order to obtain the expanded uncertainty (U), the calculated standard uncertainty was multiplied by a cover factor *k* = 2, for a level of confidence of 95%. These results showed that the recoveries for mercury in samples were within the range 91–106% (average value of 99%) of the certified values, thus being situated in the acceptable range (80–110%) according to the requirements of the Commission Decision 2002/657/EC regarding the performance criteria for quantitative methods of analysis.

Precision is usually evaluated by internal repeatability and reproducibility. In the case of Hg determination in foodstuffs, these performance parameters are imposed by requirements of the Commission Decision (2007/333/EC) and Commission Regulation (2011/836/EU) [36]. According to these regulations, the HorRat indexes are calculated as the ratio of the relative standard deviation (RSD) for repeatability (RSD_r_) or for reproducibility (RSD_R_) and the predicted relative standard deviation (PRSD) calculated using Horvitz’s equation, i.e., Equation (3) [37]. Thus, two indexes are obtained, namely, HorRat_r_ (used for repeatability conditions) and HorRat_R_ (used for reproducibility conditions). For concentrations higher than 100 μg kg^−1^, the HorRat_r_ and HorRat_R_ indexes should less than 2.

(3) $$\mathrm{PRSD}=2^{\left(1-0.5\mathrm{logC}\right)}$$

where C is equal the half of the maximum mass fraction of Hg in dietary supplements. 

According to Horvitz’s equation, the PRSD% for a concentration of 100 µg kg^−1^ (maximum admitted concentration of Hg in dietary supplements) is 23%. The relative standard deviation of repeatability was calculated by analyzing a CRM sample in six replicates using the same equipment on same day by combining the uncertainty considering the traceability chain. For RSD_R_ estimation, an intermediate reproducibility was calculated by analyzing a CRM sample in six replicates using the same equipment, but on different days, and by combining the uncertainty considering the traceability chain. The RSD_r_ was calculated to be 8.1%, while RSD_R_ was estimated to be 9.5%, meaning the HorRat indexes were 0.35% and 0.41%, respectively. These values fulfill the requirement in terms of precision for Hg determination in zeolites using TD-AAS. 

#### 2.1.5. Estimation of Measurement Uncertainty

In agreement with the requirements in Decision 2007/333/EC, the value estimated for measurement uncertainty must be lower than the maximum uncertainty of measurement (*U_f_*) estimated based on LoD value and Hg concentration in samples, using Equation (4).

(4) $$U_{f}=\sqrt{{\left(\frac{\mathrm{LoD}}{2}\right)}^{2}+{\left(\alpha *c\right)}^{2}}$$

where LoD is expressed as µg kg^−1^, c is the concentration of the maximum admitted level, and α is a numeric factor dependent on the value of the Hg concentration (α = 0.18 for concentrations of 51–500 µg kg^−1^ Hg).

According to these demands, considering the maximum level of 100 µg kg^−1^ Hg in dietary supplements, the maximum uncertainty of measurement (U_f_) should be 18 µg kg^−1^ Hg. As a consequence, the relative standard uncertainty, U_rel_, should be a maximum of 18%.

**Figure 2:**
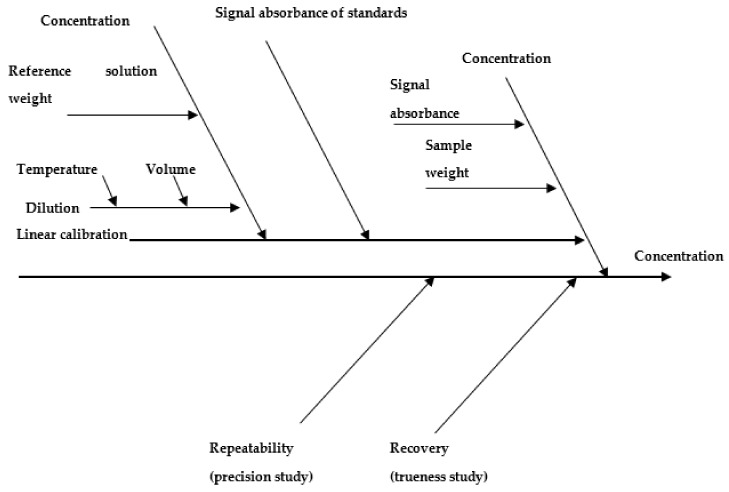
Cause and effects diagram (fishbone diagram) of uncertainties in the measurement of Hg using TD-AAS.

In our study the method had been refined from the published standard EPA Method 7473. The estimation of measurement uncertainty was based on an in-house validation process which took into account the requirements of the international standard regarding measurement uncertainty. The identified main sources of measurement uncertainty were uncertainty of calibration reference materials, uncertainty of weighted reference solutions and samples, uncertainty of the calibration curve, and accuracy and repeatability of the method, as presented in Figure 2, a cause and effects diagram (fishbone diagram). It was assumed that quality control included the total analytical procedure and has been carried out over a sufficiently long period of time and with appropriate frequency. Thus, the most significant uncertainty components related to the method can be approximated: instrument calibration, uncertainty of gravimetric operations, uncertainty of calibration standards, and uncertainty of volumetric operations will be taken into account. In this case, the main parameters affecting the measurement uncertainty of the method have been assembled into two components: trueness and precision [35].

Using these parameters, the combined standard uncertainty (*u_c_*) of the method can be calculated using the repeated measurements of CRMs, according to Equation (2).

In order to obtain a pooled uncertainty that covers the entire working range of the method, repeated measurements of CRMs with different levels of mercury were carried out. Using Equation (2), expanded uncertainty (U) for *k* = 2 was calculated for each CRM sample, with the data presented in Table 2. The pooled expanded uncertainty (%) for the method was calculated by combining the expanded uncertainties (%) of each CRM analysis, according to Equation (5), i.e.,

(5) $$U(\%)=\sqrt{\frac{U_{1}^{2}+U_{2}^{2}+U_{3}^{2}+U_{4}^{2}+U_{5}^{2}}{5}}$$

where *U* (%) is the pooled expanded uncertainty (%), while *U_1_, U_2_, U_3_, U_4_*, and *U_5_* refer to expanded uncertainties (%) found for the CRMs.

Using the formula presented in Equation (5), the value of the pooled expanded uncertainty (U%) for *k* = 2 and P = 95% was found to be 10%. This value falls well within the maximum value of 18% calculated according to the requirements in Decision 2007/333/EC, and thus is a satisfactory result.

### 2.2. Application of the TD-AAS Method to Zeolitic Tuffs Samples

#### 2.2.1. Zeolite Chemical and Mineralogical Characterization

In order to investigate the applicability of our method for mercury determination in real samples, 15 natural zeolites samples (C1–C15) from a quarry located in Chilioara, Salaj County, north-west Romania, were collected and analyzed. The samples were preliminary characterized regarding chemical composition for major elements using inductively-coupled plasma optical emission spectrometry (ICP-OES). The measured concentrations of major elements (Si, Al, Fe, Na, K, Ca, Mg, and Ti) were converted to oxides using atomic and molecular masses. The range of measured oxide concentrations is presented in Table 3.

Clinoptilolite mineral has a Si/Al ratio > 4 and dominant alkaline cations (Na + K > Ca) [38]. The content of major elements indicates that the main mineral in zeolitic tuffs collected from Chilioara deposit is K-clinoptilolite.

**Figure 3:**
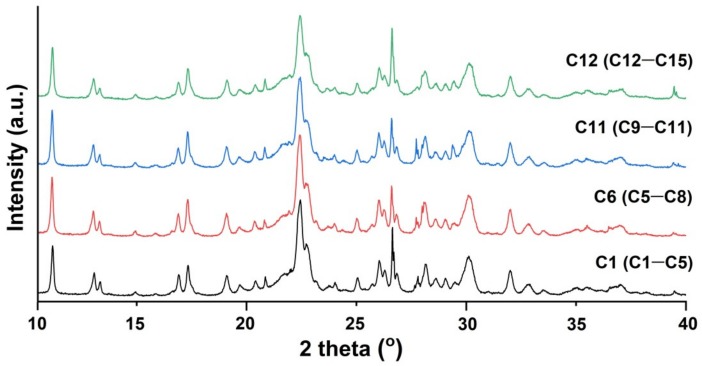
The representative XRD patterns of four zeolite groups.

According to X-ray diffraction analysis, the investigated zeolite samples (C1–C15) from the Chilioara quarry can be said to be similar and can be seen to contain up to 65% clinoptilolite and other minerals such as quartz, muscovite, feldspar, montmorillonite, and albite in lower concentrations. In Figure 3 are presented the XRD patterns of one representative sample from each group of zeolite samples (C1 from C1–C4, C6 from C5–C8, C11 from C9–C11, and C12 from C12–C15). The zeolitic tuffs samples were grouped based on their sampling points. The degree of crystallinity ranges between 65.3 and 72.8 %.

#### 2.2.2. Mercury Determination in Zeolite Samples by TD-AAS and Comparison with CV-AFS Technique

The results obtained for Hg determination in zeolite samples from the Chiloara quarry measured using the TD-AAS technique are shown in Table 4. The total Hg concentrations in the investigated samples were in the range 36.0–152 µg kg^−1^, with an average value of 69.4 µg kg^−1^. Despite the fact that in the majority of samples (80%) the concentration of Hg was below 100 µg kg^−1^, in three samples, the maximum admitted value for total Hg in dietary supplements established by legislation was exceeded. The Hg concentration varied across a relatively large domain of concentration depending on the sampling location. As a consequence, a rigorous quality control of natural zeolites used as a raw material for producing dietary supplements should exist.

In order to compare the Hg concentrations measured by TD-AAS with those measured using another technique, aliquots of same samples were digested using *aqua regia* and the concentrations of Hg from extracts were measured by CV-AFS, a widely used technique in routine laboratories. According to the significance test (t-test for dependent samples), no significant difference was found between results measured by TD-AAS and CV-AFS methods for a 95% confidence interval, regarding accuracy (t-test for dependent samples, t_calc_ = 0.904 < ttab, _ν__=14_ = 2.14).

The overall precision for Hg determination in zeolites samples, expressed as RSD%, was between 3.7 and 8.6% for concentrations measured by TD-AAS, and between 4.5 and 9.9% for measurements done by CV-AFS. The precision of the methods fulfilled the requirements imposed in Decision 2002/657/EC, giving an uncertainty of measurements lower than the limit of 18% for this concentration level.

To our knowledge, there are no literature data about the level of Hg concentration in natural zeolite samples. For soil, a world median for Hg of 50 µg kg^−1^ has been reported [39], which is considered as natural background value. The Hg concentrations measured in our study are of a similar order of magnitude. Studies on areas affected by Hg pollution due to anthropogenic activities such as mining and smelting of ores, fossil fuel combustion, and industrial chlor-alkali processes, revealed much higher Hg concentrations in soils (over 1000 µg kg^−1^) [40,41,42].

Among many minerals, zeolite is a family of microporous mineral members having a unique crystal structure providing ion exchange and adsorbent properties. Clinoptilolite is one of the most abundant natural zeolites and is recognized for its capacity to remove harmful substances like toxic metals and ammonia from different media, including human digestive system [43]. In this regard, clinoptilolite is recognized to have a positive effect on human health and thus is of high interest to be used as a dietary supplement. However, the use of clinoptilolite in human health involves strict quality control. This study is of great interest due to fact that no literature data on the Hg concentrations in natural zeolites and no standardized method to measure Hg in zeolite samples are indicated. In this regard, our study (*i*) represents a reference for future research on the Hg concentrations in natural zeolites and (*ii*) offers a fully-validated method in agreement with the requirements of legislation in the field of dietary supplements, a method that can be used by routine testing laboratories that employ the TD-AAS technique.

## 3. Materials and Methods

### 3.1. Standard Solutions, Reagents, and CRMs

A stock standard solution of Hg (1000 mg L^−1^) from Merck (Darmstadt, Germany) was used to prepare, by dilution, working standard solutions of 0.1 mg L^−1^ and 1.0 mg L^−1^, which were used for TD-AAS instrument calibration, and working standard solutions of 0.1, 0.2, 0.4, 0.6, 0.8, and 1.0 µg L^−1^, which were used for CV-AFS instrument calibration. The Hg stock standard solution used has metrological traceability to NIST SRM 3133, lot no. 061204. Multi-elemental stock standard solutions containing Na, K, Ca, Mg, Fe, and Al (1000 mg L^−1^), stock standard solutions of Si (1000 mg L^−1^), and stock standard solutions of Ti (1000 mg L^−1^) produced by Merck (Darmstadt, Germany) were used to prepare by dilution working standards solutions for ICP-OES calibration. HNO_3_ 65%, HCl 37%, and HF 40%, p.a. obtained from Merck (Darmstadt, Germany) were used for sample microwave digestion or to prepare calibration solutions. SnCl_2_·2H_2_O from Merck (Darmstadt, Germany) was used as a reductant reagent for the CV-AFS system. For all dilutions ultrapure water (18 MΩ cm^−1^) obtained from a Millipore Direct Q3 (Millipore, Molsheim, France) was used. Oxygen (4.5 quality) purchased from Linde Gas SRL Cluj-Napoca, Romania was used as a carrier gas.

Certified reference materials of soil (CRM048-50G Trace Metals Sand 1, RTC-CRM025050 Soil Sandy Loam-Metals, LGC6141 Soil contaminated with clinker ash, and LGC6135 Soil-Hackney Brick Works) and sediments (BCR 240R Lake Sediment and NCSDC78301 River Sediment) from LGC Promochem (Wesel, Germany) were used for trueness and precision studies.

### 3.2. Instrumentation and Methods

The measurements of mercury in solid samples were carried out using an Automated Direct Hg Analyzer Hydra-C (Teledyne Instruments, Leeman Labs, Mason, OH, USA), an instrument that includes a furnace module based on thermal desorption of analyte from the sample and a measuring module which is based on the atomic absorption spectrometry principle. To measure the Hg content, the sample is weighted in nickel boats, which are introduced into the furnace module. Here the sample is dried and decomposed at a temperature that assures the releasing of Hg along with other compounds. All these compounds are carried through a catalyst tube by an oxygen flow, where Hg^2+^ is oxidized to metallic mercury (Hg^0^). The catalyst tube also retains interfering compounds like halogens and nitrogen/sulfur oxides. The final flow passes through a gold amalgamator which collects Hg^0^. After this stage, the amalgamator is heated, thus releasing mercury that is carried to the atomic absorption spectrometer module. This contains two measuring cells, a high sensitivity cell and a low sensitivity cell, where the mercury vapors are introduced. The absorbance signal at a wavelength of 253.65 nm emitted by an Hg lamp was measured in series in the two cells. Instrument calibration was accomplished using aqueous standards prepared in 5% HNO_3_. Working standards were blank, 0.1 mg L^−1^, and 1.0 mg L^−1^ at six different injection weights for each calibration ranges (high and low sensitivity). The calibration curves plotted the micro-absorbance versus the amount of total mercury injected.

The content of major elements was determined by ICP-OES using an Optima 5300 DV (Perkin Elmer, Woodbridge, ON, Canada). Microwave digestion of a 0.25 g sample was done with a mixture of 3 mL HNO_3_ 65%, 9 mL HCl 37%, and 2 mL HF 40% in a closed-vessel MWS-3+ microwave system (Berghof, Eningen, Germany). Polytetrafluoroethylene (PTFE) digestion vessels were pre-cleaned with 10% (*v*/*v*) HNO_3_ for 24 h to avoid contamination. 

The mineralogy and the crystallinity of zeolite samples were investigated using a Bruker D8 Advance diffractometer (Karlsruhe, Germany) using CuKα radiation (λ = 1.54056 Å) at 40 kV and 40 mA. The data were collected in the 2θ range 10–40°, with a step size of 0.02° and a counting time of 0.5 s per step.

A cold vapor atomic fluorescence spectrometer Hydra-AF (Teledyne Instruments, Leeman Labs, Mason, OH, USA) was used for Hg determination from digested samples. For wet digestion of zeolite samples, a closed-vessel microwave system Berghof MWS-3^+^ with temperature control mode (Berghof, Eningen, Germany) was used. To 1 g of powdered zeolite sample, 9 mL HNO_3_ 65% and 21 mL HCl 37% were added for digestion. After cooling at room temperature, the slurry was diluted to 100 mL with ultrapure water and then filtered through 0.45 μm cellulose membrane filters. Digested samples were analyzed using the CV-AFS method. Hydra AF is a continuous flow system where sample and reductant, in this case 2% SnCl_2_·2H_2_O in 3.6% (*v*/*v*) HCl ultrapure, are pumped into a gas/liquid separator. There the mercury in the sample is reduced to elemental mercury, being carried as a gas to the spectrometer. The instrumental parameters employed for CV-AFS determinations are presented in Table 5.

### 3.3. Zeolitic Tuff Sample Collection and Preparation

Zeolitic tuff samples were collected in July 2019 from Chilioara, Salaj County, north-west Romania. In this region, the predominant zeolite in tuff is represented by clinoptilolite-type minerals and was formed in a marine environment in the Miocene and Pliocene periods from volcanic glass, magmatic quartz, micas, and silicates [44]. The samples were collected as rock from the Chilioara quarry, where they were crushed and further grounded to a fine powder in a tungsten-carbide swing mill and sieved through a 100 µm mesh sieve. The fraction below 100 µm was further homogenised by mixing in a PVC drum for 1 h, after which the samples were stored in closed bottles at room temperature until analysis. 

### 3.4. Strategy for Method Validation

Method validation was accomplished by assessing the main performance parameters: selectivity, linearity, LoD, LoQ, working range, trueness, precision, and measurement uncertainty. LoD, LoQ, precision, and accuracy were evaluated considering the requirements of European legislation for official control of mercury in dietary supplements (Decisions 2002/657/EC, 2007/333/EC, and 2011/836/EC) [26,33,36].

## 4. Conclusions

In this work, it has been demonstrated that an analytical method based on the TD-AAS technique can be successfully used for determination of low Hg concentrations in zeolites. The method was validated considering the requirements of European legislation for official control of Hg in dietary supplements. Compared with other methods used for mercury determination in solid samples this technique is fast since no previous sample digestion is required. In addition, because no mineral acids are necessary for sample digestion, the study is in the current trend of green chemistry for the development of environmentally-friendly methods. The results obtained for LoQ and other figures of merit such as trueness, precision, and measurement uncertainty found by the analysis of certified reference materials validate agreement of the method with the requirements of legislation in this field. The paper presents all the stages required to validate the method of Hg determination in zeolites using TD-AAS.

## Figures and Tables

**Table 1:** Instrumental settings for Hg determination using the thermal desorption atomic absorption spectrometry (TD-AAS) system.

Parameter	Setting
Dry	300 °C for 30 s
Decomposition	850 °C for 200 s
Catalyst	600 °C
Catalyst wait period	60 s
Gold trap	700 °C for 30 s
Measurement time	90 s
Oxygen flow rate	300 min^−1^

**Table 2:** Results for total Hg in certified reference materials (CRMs) analyzed by TD-AAS.

CRM	Certified Values ± U ^a^ (mg kg^−1^)	Measured Values ± U ^b^ (mg kg^−1^)	Recovery ± U ^a,b^ (%)
	Total Hg	Total Hg	Total Hg
CRM048-50G	28 ± 1.13	28.2 ± 0.93	101 ± 3
RTC-CRM025050	99.8 ± 31.7	106 ± 7.2	106 ± 7
LGC6135	3.2 ± 0.4	3.3 ± 0.33	103 ± 10
BCR 240R	1.46 ± 0.2	1.4 ± 0.15	96 ± 11
NCSDC78301	0.22 ± 0.04	0.20 ± 0.03	91 ± 15

^a^—U is expanded uncertainty for *k* = 2 and 95% confidence level; ^b^—*n* = 6 parallel determinations.

**Table 3:** Major oxides concentrations (%) in zeolite samples from the Chilioara deposit, north-west Romania.

Compounds	Range of Concentrations (%)
SiO_2_	67.3–68.9
Al_2_O_3_	9.55–11.1
CaO	2.05–2.99
MgO	0.42–0.84
K_2_O	1.88–2.46
Na_2_O	0.39–1.11
TiO_2_	0.19–0.22
Fe_2_O_3_	0.78–1.32

**Table 4:** Concentrations of total Hg (µg kg^−1^) measured in zeolite samples from the Chilioara deposit by TD-AAS and cold vapor atomic fluorescence spectrometry (CV-AFS) methods (*n* = 3 parallel measurements). Legend: RSD, relative standard deviation.

Zeolite Sample	TD-AAS		CV-AFS	
	Average ± st. dev. (µg kg^−1^)	RSD (%)	Average ± st. dev. (µg kg^−1^)	RSD (%)
C1	56.2 ± 2.88	5.1	54.6 ± 4.12	7.6
C2	122 ± 4.50	3.7	116 ± 5.22	4.5
C3	44.7 ± 3.26	7.3	48.2 ± 3.02	6.3
C4	112 ± 5.22	4.7	117 ± 7.43	6.4
C5	88.4 ± 6.25	7.1	86.3 ± 5.99	6.9
C6	36.5 ± 3.15	8.6	34.9 ± 2.77	7.9
C7	36.0 ± 2.22	6.2	36.2 ± 2.53	7.0
C8	77.6 ± 3.88	5.0	77.8 ± 4.36	5.6
C9	54.8 ± 3.15	5.7	57.9 ± 5.33	9.2
C10	63.6 ± 4.22	6.6	62.1 ± 4.69	7.6
C11	43.3 ± 2.89	6.7	40.6 ± 4.01	9.9
C12	40.0 ± 2.67	6.7	39.8 ± 3.54	8.9
C13	72.5 ± 2.68	3.7	72.7 ± 6.25	8.6
C14	152 ± 6.17	4.1	147 ± 9.62	6.5
C15	42.0 ± 2.22	5.3	40.1 ± 3.57	8.9
Average	69.4	5.8	68.8	7.5

**Table 5:** Instrumental settings for Hg determination using the CV-AFS system.

Parameter	Setting
Argon flow-rate	700 mL min^−1^
Sample flow-rate	5 mL min^−1^
Reductant flow-rate	1 mL min^−1^
Uptake time	25 s
Rinse time	60 s
Integration time	10 s
